# 542. Use of Bamlanivimab in Cancer Patients with Mild-to-Moderate COVID-19

**DOI:** 10.1093/ofid/ofab466.741

**Published:** 2021-12-04

**Authors:** Patricia Brock, Hiba Dagher, Adriana H Wechsler, Demis N Lipe, Patrick Chaftari, Anne-Marie Chaftari, Maria S Gaeta, Tami N Johnson, Daniel J Coussirat, Samuel L Aitken, Samuel L Aitken, Ying Jiang, Alexandre Malek, Ray Y Hachem, Issam I Raad

**Affiliations:** 1 UT MD Anderson Cancer Center, Houston, TX; 3 The University of Texas MD Anderson Cancer Center, Houston, TX; 4 Michigan Medicine, Ann Arbor, TX; 5 MD Anderson Cancer Center, Houston, TX

## Abstract

**Background:**

Bamlanivimab is a monoclonal antibody that was granted an emergency use authorization by the US Food and Drug Administration in November 2020 for patients with mild to moderate coronavirus disease 2019 (COVID-19). It initially showed promising results with decreasing hospitalizations and return emergency department visits in immunocompetent patients. We evaluated the role of bamlanivimab in the cancer patient population.

**Methods:**

We conducted a retrospective matched study of all cancer patients diagnosed with mild to moderate COVID-19 who received bamlanivimab in our acute cancer care center (ACCC) from December 2020 to February 2021. These patients were compared to a control group of cancer patients who presented to our ACCC and were diagnosed with mild to moderate COVID-19 from March to November 2020 before the introduction of bamlanivimab. Control patients were matched by age and underlying malignancy. All patients had a baseline oxygen saturation ≥ 94% and an absolute neutrophil count > 500 mm^3^. Demographics, clinical characteristics, and outcome that included COVID-related admissions, oxygen desaturation, ICU admission and 30-day mortality were compared in both groups.

**Results:**

A total of 108 patients were analyzed with 54 patients in each group, of which 59% consisted of hematologic malignancies, and 33% were ≥ 65 years. The presenting symptoms were similar in both groups and mainly consisted of cough, fever, and dyspnea. Patients who received bamlanivimab were less likely to be admitted to the hospital (24% vs. 91%; p< 0.0001), experience oxygen desaturation < 94% during follow-up (11% vs 44%; p=0.0001), require oxygen supplement (7% vs. 44%; p< 0.0001), or be admitted to the ICU (4% vs 15%; p=0.046). No 30-day mortality was observed in the bamlanivimab group with 2 (4%) occurring in the control group. However, the difference was not significant.

**Conclusion:**

Bamlanivimab decreased hospital and ICU admissions in cancer patients. In addition, bamlanivimab reduced oxygen requirement and the risk of hypoxia and progression to severe disease in this patient population.

**Disclosures:**

**Samuel L. Aitken, PharmD, MPH, BCIDP**, Melinta Therapeutoics (Individual(s) Involved: Self): Consultant, Grant/Research Support

